# Responding to Natural and Industrial Disasters: Partnerships and Lessons Learned

**DOI:** 10.1017/dmp.2020.467

**Published:** 2021-03-16

**Authors:** Elaine Symanski, Heyreoun An Han, Inkyu Han, Michelle McDaniel, Kristina W. Whitworth, Sheryl McCurdy, William Brett Perkison, Amal Rammah, P. Grace Tee Lewis, George L. Delclos, Elena Craft, Melissa Bondy, Cheryl Lyn Walker, Loren Hopkins, José Guillermo Cedeño Laurent, Daisy James

**Affiliations:** 1 Center for Precision Environmental Health, Department of Medicine, Baylor College of Medicine, Houston, Texas, USA;; 2 Gulf Coast Center for Precision Environmental Health, Baylor College of Medicine, Houston, Texas, USA;; 3 Southwest Center for Occupational and Environmental Health (SWCOEH), The University of Texas Health Science Center at Houston (UTHealth) School of Public Health, Houston, Texas, USA;; 4 Center for Health Promotion and Prevention Research, UTHealth School of Public Health, Houston, Texas, USA;; 5 Environmental Defense Fund, Austin, Texas, USA;; 6 Department of Epidemiology and Population Health, Stanford University, Stanford, California, USA;; 7 Center for Precision Environmental Health, Departments of Molecular & Cell Biology and Medicine, Baylor College of Medicine, Houston, Texas, USA;; 8 Houston Health Department, Houston, Texas, USA;; 9 Department of Statistics, Rice University, Houston, Texas, USA; 10 Department of Environmental Health, Harvard T.H. Chan School of Public Health, Boston, Massachusetts, USA

**Keywords:** disaster response, natural disasters, industrial fire, public health, partnerships

## Abstract

**Objectives:**

The aim of this study was to provide insights learned from disaster research response (DR2) efforts following Hurricane Harvey in 2017 to launch DR2 activities following the Intercontinental Terminals Company (ITC) fire in Deer Park, Texas, in 2019.

**Methods:**

A multidisciplinary group of academic, community, and government partners launched a myriad of DR2 activities.

**Results:**

The DR2 response to Hurricane Harvey focused on enhancing environmental health literacy around clean-up efforts, measuring environmental contaminants in soil and water in impacted neighborhoods, and launching studies to evaluate the health impact of the disaster. The lessons learned after Harvey enabled rapid DR2 activities following the ITC fire, including air monitoring and administering surveys and in-depth interviews with affected residents.

**Conclusions:**

Embedding DR2 activities at academic institutions can enable rapid deployment of lessons learned from one disaster to enhance the response to subsequent disasters, even when those disasters are different. Our experience demonstrates the importance of academic institutions working with governmental and community partners to support timely disaster response efforts. Efforts enabled by such experience include providing health and safety training and consistent and reliable messaging, collecting time-sensitive and critical data in the wake of the event, and launching research to understand health impacts and improve resiliency.

Houston Texas is vulnerable to natural disasters, including floods and hurricanes, as well as industrial disasters, which can lead to adverse exposures to chemical, biological, physical, and psychosocial stressors. In August 2017, Hurricane Harvey was the wettest tropical cyclone to impact the United States with over 50 inches of rain and record-breaking catastrophic flooding along the Gulf Coast. More than 30,000 residents were affected and over 200,000 homes destroyed at an estimated cost of $130 billion.^1^

In response to Hurricane Harvey, the Southwest Center for Occupational and Environmental Health (SWCOEH) at The University of Texas Health Science Center at Houston (UTHealth) teamed with academic, community, and governmental partners to launch disaster research response (DR2) activities and address environmental health concerns in affected communities. These DR2 studies laid the groundwork for a response to an industrial fire 18 mo later at the Intercontinental Terminals Company (ITC), a storage facility for petroleum liquids and gases in Deer Park, Texas, a suburb of Houston.^2^ In this study, we describe our action-to-research disaster response to Hurricane Harvey and the ITC fire and provide lessons about how academic institutions can use their resources and infrastructure to partner with community groups and governmental agencies to improve disaster resiliency.

## Methods

All activities were approved by UTHealth’s Committee for the Protection of Human Subjects and the Institutional Review Boards (IRBs) at participating institutions.

Following Hurricane Harvey’s historic landfall, an Incident Command structure was put in place. The UTHealth Harvey response team secured funding and coordinated activities with external partners, including colleagues at Baylor College of Medicine (BCM), Harvard T.H. Chan School of Public Health, Houston Health Department (HHD) and Environmental Defense Fund (EDF). Our disaster response actions included environmental monitoring, distributing safety kits, providing training on proper cleanup of flooded homes and businesses, gathering oral histories, developing an informational website, and hosting worker safety seminars. We reached out to religious leaders and community partners of an ongoing community-engaged research project^3^ to identify neighborhoods for environmental sampling that were most affected by the storm as well as participated in community events to deliver in-person trainings on cleanup safety. Led by BCM colleagues, we assisted in developing and launching the Houston Hurricane Harvey Health (Houston 3H) DR2 study to evaluate the impacts of the storm on mental and physical health.^4^ Participants completed self-administered questionnaires about demographics, flood-related exposures, and health outcomes; provided nasal, oral, and fecal samples for microbiome analyses; and wore silicone wristbands as passive personal samplers to assess exposure to over 1500 chemicals.

## Results

In total, 71 volunteers were trained to conduct environmental monitoring and provide health and safety education to residents and workers involved in cleanup of flooded homes and businesses. We attended 19 community events and presented in-person, bilingual safety training sessions where we distributed approximately 1200 safety kits. Six months later, we re-contacted over 80 individuals who had received the safety training and assessed their health, the status of remediation efforts, and the effectiveness of the training.^5^ Also, approximately 60 public health officials, faculty, staff, and students and nonprofit organization (eg, *Fe y Justicia* Worker Center) members participated in sponsored disaster response training events. Within 3 months of Harvey’s landfall, 201 soil samples and 38 water samples were collected from Houston-area neighborhoods and analyzed for 24 metals. RESCUE real-time air sensors were installed in 12 homes (10 flooded homes with on-going remediation and 2 nonflooded homes) for a 6-month period. Over 100 oral histories were gathered from Muslim leaders who opened their mosques to non-Muslim flood victims; social workers at Texas Children’s Hospital in Houston who worked or volunteered during the storm; Cajun Navy volunteers; entrepreneurs in Dallas who made T-shirts to raise funds for Harvey victims; first responders and emergency response coordinators; and mothers and teachers from schools in Houston’s low-income neighborhoods. Also, within 30 days of the storm making landfall, 208 participants were enrolled in the Houston 3H study from 4 sites across Harris County.^4^

Learnings from these immediate post-Harvey activities included experience with publicly available resources (eg, Rapid Acquisition of Pre- and Post-Incident Disaster Data (RAPIDD) protocols) available within the DR2 community; development of protocols for recruiting, training, and scheduling student volunteers; and development of adaptable in-house field training materials, disaster protocols, and environmental/biological monitoring procedures. These efforts also increased the familiarity of IRB with the rapid approval needed in a disaster setting and established multi-directional avenues for communication with community members and other stakeholders.

**Figure 1.**
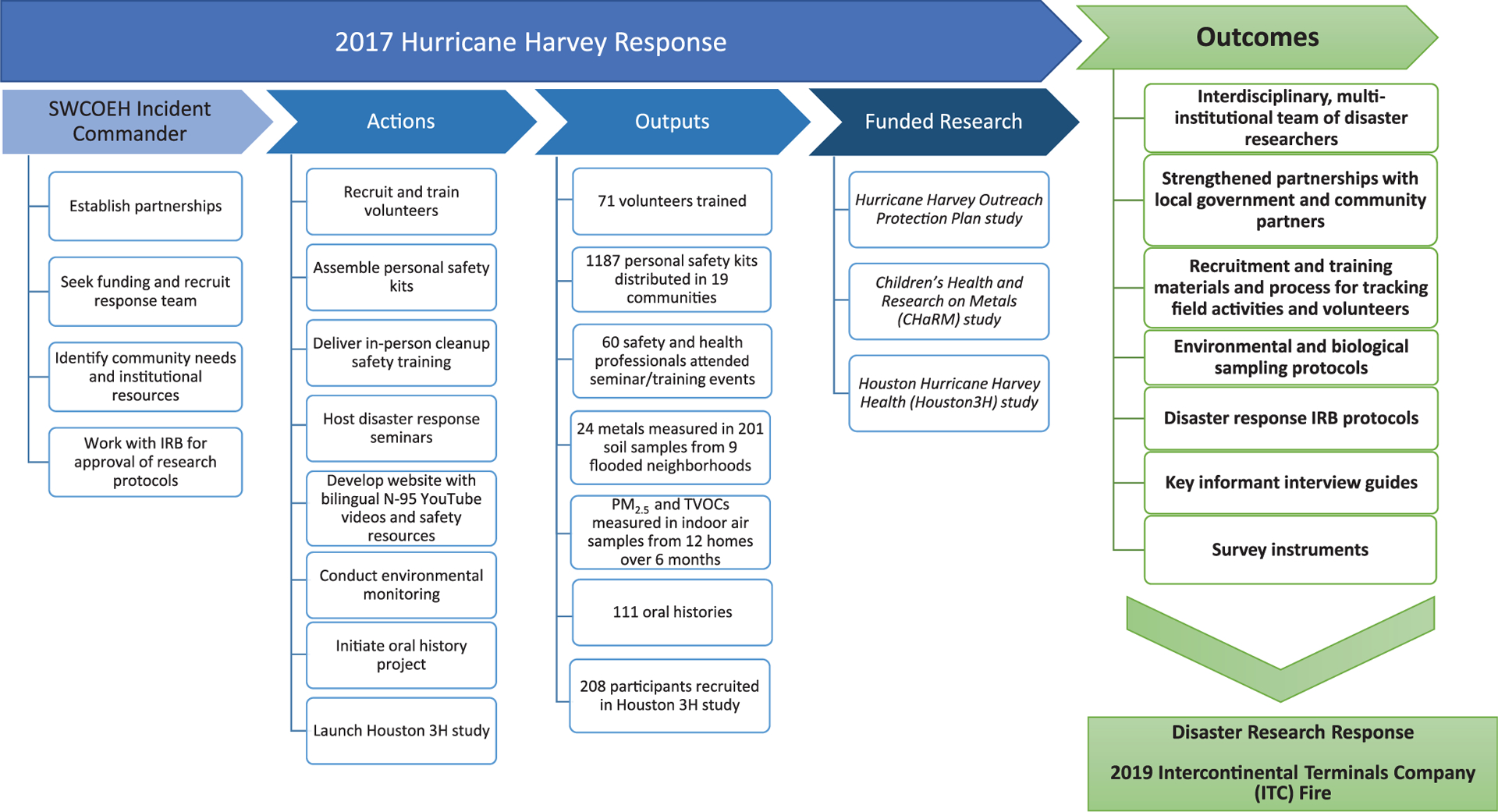
2017 Hurricane Harvey response and outcomes.

Drawing on these lessons from our multi-faceted response to Hurricane Harvey, and the partnerships established as a result of those efforts (Figure 1), we quickly mobilized to address concerns related to increased air pollutant exposures from the 6-d ITC fire that started on March 17, 2019. This fire resulted in community evacuations, shelter-in-place orders, and closures of school districts in the surrounding area. Teams of researchers, now familiar with rapid deployment approaches were able to train and deploy student volunteers rapidly to monitor black carbon, ultra-fine particles, fine particulate matter (PM_2.5_) and total volatile organic compounds (TVOCs) while the fire was burning. Experts trained student volunteers on sampling procedures and code of conduct in the field by modifying the protocols developed during Hurricane Harvey DR2 efforts.

Based on our experience gaining rapid approval for Hurricane Harvey activities, our IRB was better positioned to provide expedited approval for a DR2 study in response to the ITC fire, the Deer Park Chemical Fire (DeeP Fire) study.^2^ Furthermore, the Harvey study protocols served as preplanned and in-house templates that were adapted for developing recruitment strategies, informed consents, health questionnaires, and in-depth interview probes. Faculty and trained student volunteers conducted health surveys and in-depth interviews. They also installed RESCUE air sensors, which were being stored in-house following Hurricane Harvey, for monitoring of ambient air PM_2.5_ and TVOCs at resident homes over 6 weeks. Furthermore, we co-located passive samplers for analysis of 31 VOCs. Prior decisions about reporting back results to study participants in the Houston 3H study facilitated plans for reporting individualized results of residential concentrations of the monitored air pollutants. Drawing on enriched collaborations following Hurricane Harvey, we assisted EDF and HHD in installing 20 Clarity solar-powered and weatherproof real-time air sensors to monitor PM_2.5_ and nitrogen oxides outdoors at schools, homes, and fire departments to establish an air quality surveillance network in Deer Park and surrounding areas.

## Discussion

In the face of these two disasters that affected the Texas Gulf Coast, we were able to apply what we learned from one disaster to the second to mobilize volunteers quickly, tap into our infrastructure, and work with colleagues to address community needs and launch DR2 activities. While the SWCOEH had a commitment to, and long-standing history of, research, outreach, and service, particularly among vulnerable populations and workers, other partners new to DR2 activities gained valuable experience during Harvey applicable in other disaster settings, including the ITC fire. Responding to Hurricane Harvey required protocols with a much shorter response time and “boot-strapping” activities with more limited resources than previous efforts. However, what was learned in this process—even though the disasters were quite different—provided a strong foundation for our response to the ITC fire 18 months later.

Preplanning (that we had not done before Hurricane Harvey) facilitated identification of local expertise and capabilities, within and across institutions, which enabled a more effective response to the ITC fire. Such preplanning requires drawing on the DR2 experience, resources, and mechanisms already available,^6^ having in-house environmental sampling equipment, and developing IRB disaster research protocols that could be tailored for different types of emergencies. For example, we were able to respond more quickly and deploy an air monitoring team in the field within 48 hr of the ITC fire. Furthermore, we launched a DR2 study by implementing protocols that we developed following Hurricane Harvey (e.g., for recruiting, training, and scheduling volunteers; for conducting key informant interviews; and administering health surveys). Key to our response to both disasters was working with an interdisciplinary team of academics as well as partners from the government and the community to enable meaningful science.^7^ Moving forward, there is also need for continued support of DR2 toolkits currently available and support from academic institutions to facilitate response efforts to disasters in the future.

The use of student volunteers is recognized as a valuable resource in communities facing disasters. By providing on-the-ground training, students have an opportunity for service learning through experiential education rooted in action in the community. One strategy we used following Harvey was to tap into an ongoing course by developing a project focused on collecting oral histories from individuals impacted by the storm or involved in response or recovery efforts. Furthermore, we trained student volunteers in environmental sampling and survey administration following both disasters. Having already completed human subjects training facilitated and accelerated their participation in these activities. However, a challenge was working with students in providing training and scheduling field visits, the former of which focused on emphasizing commitment and responsibilities and the latter of which required frequent email exchanges to stay abreast of changing schedules. Establishing on-going coursework or training on disaster response in college curricula would increase student capacity when disasters occur.

Following Hurricane Katrina, the inequitable distribution of Hurricane-related flooding effects was framed for the first time within the context of environmental justice.^8^ Subsequently, Collins et al.^9^ applied this framework to post-Harvey Houston and found that households of color and low socioeconomic status (SES) experienced more extensive flooding than non-Hispanic whites and higher SES households. Recognizing cultural differences in working with low income and diverse ethnic/racial communities (41% of Harris County residents and over half of the county’s population living in poverty are Hispanic^10^), we prepared our safety information, videos, and questionnaires in Spanish as well as English. However, we faced difficulty in identifying enough bilingual field team members during both disasters who could assist in communicating with Spanish-speaking individuals. This could be partially remediated in the future in strengthening partnerships with community partners that have staff fluent in Spanish. Houston’s diverse population speaks 145 languages,^11^ so expanding to other languages beyond English and Spanish is also important.

Many of our initial activities in response to Harvey and the ITC fire were driven by our commitment to serve neighborhoods in need. In distributing cleanup safety kits to residents and workers, we hoped that they would make the connection between environmental exposures and health and promote changes in behavior to remediate flooded homes and businesses. In the DR2 studies that we conducted, most participants were interested in receiving their individual results, which created additional opportunities for informal learning about environmental health.^12^ In working with local public health officials following Harvey, we developed an informational webpage with HHD, and we communicated our real-time air monitoring results to the Harris County Public Health, which also engaged in assessing community air quality during and after the ITC fire. We later participated in a workgroup convened by Harris County Public Health that led to a commitment of over $11 million to expand staffing and resources to improve the county’s response to disasters.^13^

Unfortunately, a major challenge that we and others face in addressing health effects related to environmental exposures following catastrophic events is not having collected baseline exposure data before a disaster. As shown in Table 1, given the disaster trajectory in the Greater Houston area, it is not a question of *if* Houston will experience another disaster but rather *when*. Hence, we expect the data we collected post-Harvey and post-ITC fire will provide valuable baseline information when the next disaster hits.

## Conclusions

Response, recovery, and research efforts have laid the groundwork for improving our capacity for timely and efficient response to disasters. Challenges ahead include sustaining collaborations with partners and enhancing infrastructure that will facilitate future DR2 efforts. It is incumbent upon academic institutions to work together locally and nationally, and with public health agencies and other governmental and community partners, to support time-critical outreach, education, training, and research to address the needs of communities impacted by disasters.

## Figures and Tables

**Table 1.** Notable natural and industrial disasters in the greater Houston area since 1994

Date	Natural or industrial disaster	Date	Natural or industrial disaster
Oct-94	Hurricane Rosa	Jan-16	Marathon Petroleum Corporation refinery fire
Oct-94	Pipeline explosion	Mar-16	Pasadena Refinery System, Inc. explosion
Jul-95	Houston Distribution Inc. warehouse fire	Apr-16	Tax Day Flood
Sep-98	Tropical Storm Frances	Apr-16	ExxonMobil plant fire
Nov-98	Storms cause heavy flooding	Apr-16	Lyondell Bassell plant fire
Jun-01	Tropical Storm Allison	May-16	Custom Packaging and Filling Company fire
Aug-02	Houston Fuel Oil Terminal Co. fuel tank fire	May-16	Memorial Day Flood of 2016
Jun-03	Nova Chemicals plant fire	Aug-17	Hurricane Harvey
Mar-05	BP America refinery explosion	Apr-18	TPC Group chemical storage tank fire
Jun-06	Torrential rainfall causes flooding	Apr-18	Valero Energy plant fire
Sep-08	Hurricane Ike	May-18	Kuraray America EVAL plant fire
Apr-09	Heavy rainfall & extensive flooding	Mar-19	ExxonMobil plant fire
Dec-09	American Acryl plant explosion	Mar-19	ITC chemical fire
Jul-12	Heavy rainfall causes flooding	Sep-19	Tropical Storm Imelda
Aug-14	Rainstorms flood Greens Bayou	Nov-19	TPC Group plant explosion
May-15	Memorial Day Flood of 2015	Jan-20	Watson plant explosion
